# Trends in Proportion of Delirium Among Older Emergency Department Patients in South Korea, 2017–2022

**DOI:** 10.5811/westjem.41507

**Published:** 2025-11-26

**Authors:** Jeongmin Moon, Seonji Kim, Daesung Lim, Ho Kyung Sung, Nami Lee, Kyung-Shin Lee

**Affiliations:** * Research Institute for Public Healthcare, National Medical Center, Seoul, Republic of Korea; † Yonsei University College of Medicine, Department of Biomedical Systems Informatics, Seoul, Republic of Korea; ‡ Institute for Innovation in Digital Healthcare, Yonsei University, Seoul, Republic of Korea; § Seoul Medical Center, Department of Emergency Medicine, Seoul, Republic of Korea; ¶ Seoul National University, College of Medicine, Department of Human Systems Medicine, Seoul, Republic of Korea; || National Medical Center, National Emergency Medical Center, Seoul, Republic of Korea; # Seoul National University Hospital, Department of Public Health, Seoul, Republic of Korea; ** Center for Public Healthcare Policy, National Medical Center, Seoul, Republic of Korea

## Abstract

**Introduction:**

Delirium is a critical neuropsychiatric condition that surged among older adults during the coronavirus disease 2019 **(**COVID-19) pandemic, likely due to social isolation resulting from distancing measures. In this study we examined trends in delirium-related emergency department (ED) visits before and during the pandemic using nationwide data from South Korea, with a focus on different phases of social distancing, to inform healthcare strategies for older adults during public health crises.

**Methods:**

We obtained data from the National Emergency Department Information System (2017–2022). Changes in ED visits were assessed across pre-pandemic (January 2017–January 2020), early pandemic (February 2020–March 2022), and late pandemic (April 2022–December 2022) phases using interrupted time series analysis.

**Results:**

A total of 80,442 delirium-related ED visits among adults ≥ 65 years of age were recorded. The interrupted time series analysis showed a significant step increase in ED visits during the early pandemic phase (relative risk [RR] 1.290, 95% CI 1.201–1.386; 29.0% increase), followed by a decrease in the late pandemic phase (RR 0.922, 95% CI 0.868–0.981; 7.8% decrease). The most substantial increase was for individuals 65–74 year of age during the early pandemic period (RR 1.406, 95% CI 1.264–1.564) reflecting a 40.6% increase in visits to the ED. Indirect ED visits, such as institutional referrals, also notably increased (RR 1.275, 95% CI 1.184–1.373) reflecting a 27.5% increase.

**Conclusion:**

Delirium-related ED visits among older adults showed a notable 7.8% decrease during the late pandemic period, with key risk groups identified, particularly adults 65–74 of age (40.6% increase) and those referred from institutions (27.5% increase) during the early pandemic period. These findings may help inform targeted interventions and public health responses in similar healthcare settings. Despite limitations including reliance on diagnostic codes, lack of subgroup analysis by COVID-19 status, potential duplicate visit counts, and limited regional granularity this study offers important insight into delirium care needs during crisis periods. Further research should further explore causal mechanisms and the specific impact of COVID-19 infection on delirium incidence.

## INTRODUCTION

Delirium is a critical neuropsychiatric condition marked by disruptions in attention and consciousness, leading to a rapid decline in cognitive function.^1^ It often results from underlying medical issues such as infections, metabolic imbalances, or medication effects.^2^ If not promptly recognized and managed, delirium can lead to significant morbidity and mortality. Delirium occurs in approximately 7–10% of older emergency department (ED) patients,^3^ with research indicating rates as high as 10–30%.^2^ Furthermore, delirium affects up to 50% of hospitalized older adults, particularly among those who initially presented to the ED, underscoring the importance of addressing this issue in both hospital and community settings.^4^

The coronavirus disease 2019 **(**COVID-19) pandemic has drawn increased attention to the incidence of delirium among older patients, particularly in emergency and inpatient settings. A systematic review and meta-analysis demonstrated that delirium was highly prevalent among older adults with COVID-19, significantly elevating their risk of mortality.^5^ Several factors contributed to the rising incidence of delirium during the pandemic, including overwhelmed healthcare systems, staff shortages, and stringent infection-control measures. The multifactorial nature of COVID-19-associated delirium, which may result from severe respiratory distress, systemic inflammation, and the effects of prolonged social isolation, further complicates its management.^6^–^8^ Studies conducted in Japan and Germany highlighted how visitation restrictions and patient isolation during the pandemic escalated delirium rates among hospitalized COVID-19 patients, particularly among older adults.^9^,^10^ Analyzing factors such as age, housing, and hospitalization is crucial for both healthcare and public policy.

The pandemic exacerbated the vulnerabilities of older patients, revealing significant gaps in the ability of healthcare systems to effectively manage and prevent delirium.^11^ These challenges encountered during the COVID-19 crisis underscore the need for improved protocols, increased staffing, and heightened awareness of delirium as a critical condition, particularly in emergency settings.^12^ However, because most research has focused on Western countries, comprehensive studies on the impact of COVID-19 on delirium in South Korea are lacking. During the COVID-19 pandemic, the government of South Korea implemented stringent public health interventions, including centralized triage protocols, nationwide social distancing, and restricted hospital visitation policies, which likely influenced patterns of ED use among older adults.^13^

Notably, a South Korean study examining the effects of intensive care unit (ICU) visitation policies during the pandemic found no difference in overall delirium incidence between the restricted and non-visitation groups. However, significant differences in delirium subtypes and anxiety levels were observed. Patients without visitors were more likely to experience hyperactive and mixed delirium and had significantly higher anxiety levels, underscoring the potential role of family presence in the management of delirium during crisis situations.^14^ Cultural differences, such as family involvement in older adult care, improvement in healthcare infrastructure, and implementation of various public health strategies, may significantly reduce the incidence and promote better management of delirium among older patients.^15^,^16^

**Population Health Research Capsule**

What do we already know about this issue?

*Delirium is common in older ED (emergency department) patients and may worsen during public health crises due to social isolation and disrupted healthcare access.*

What was the research question?

*How did delirium-related ED visits among older adults change before and during COVID-19 in South Korea?*

What was the major finding of the study?

*Delirium ED visits rose 29.0% early in the pandemic (RR 1.290, 95% CI 1.201–1.386) and fell 7.8% later (RR 0.922, CI 0.868–0.981).*

How does this improve population health?

*Findings highlight the need for targeted delirium monitoring and support strategies for older adults during health crises to reduce ED burden.*

In this study we aimed to examine temporal trends in delirium presentations to EDs before and during the COVID-19 pandemic, with the goal of understanding how the pandemic and associated public health measures may have affected delirium incidence and healthcare use among older adults. By identifying changes in delirium patterns, we sought to inform future preparedness and response strategies for vulnerable populations in times of public health emergencies. Rather than examining delirium occurrence among confirmed COVID-19 patients, we sought to explore how broader pandemic-related societal measures, including social distancing and restricted healthcare access, influenced temporal trends in delirium presentations in EDs. This approach was intended to capture the wider public health impact on older adults beyond the effects of direct infection.

## METHODS

### Data Source

We constructed a cross-sectional study using data from the National Emergency Department Information System (NEDIS). This observational study follows the Strengthening the Reporting of Observational Studies in Epidemiology reporting guidelines. The NEDIS compiles real-time patient information from more than 400 EDs across South Korea.^17^ It is a government-operated emergency information network managed by the Ministry of Health and Welfare and the National Emergency Medical Center. Emergency centers across the country undergo annual evaluations to maintain their status as emergency service institutions. As part of this process, they are required to digitize all NEDIS data and submit the information for assessment. The NEDIS database encompasses a range of demographic and clinical information for all patients who visited EDs. Detailed information has been presented in previous studies.^18^–^20^

### Study Population

Patients ≥ 65 years of age who visited EDs between January 1, 2017–December 31, 2022 were included in the study. Variables considered included age, sex, region, and insurance status, along with detailed clinical data related to ED visits, such as arrival and departure times and dates, mode of arrival, transport method, chief complaints, and Korean Triage Acuity Scale (KTAS) scores. We also recorded patient disposition and diagnosis codes based on the *International Classification of Diseases*, *10^th^ Ed*, (ICD-10). We categorized EDs into three levels based on hospital function and capacity, with data collection protocols varying by ED category.

### Study Outcomes and Variables

The selection of study outcomes and variables was guided by a review of established literature on delirium risk factors in older adults. In particular, Inouye et al (2014) provided a comprehensive framework for identifying key risk factors such as infections, metabolic disturbances, and medication use, which informed the classification of comorbid conditions included in our analysis.^4^ The primary outcome was delirium, as defined using ICD-10 codes (F05.0–F05.1, F05.8–F05.9). The ICD-10 codes were more frequently recorded for hyperactive or mixed delirium (42.9%) than for hypoactive delirium (14.3%) or normoactive delirium (5.9%).^21^ The other outcomes of interest were predisposing factors for delirium, defined as diagnoses or treatment codes based on ICD-10 or Unified Medical Language System codes, detailed in Supplementary Table 1, with their categorization based on previous studies.^1^,^9^,^20^,^22^,^23^ We grouped and analyzed these diagnosis categories to evaluate changes in the distribution of predisposing medical conditions for delirium across the pandemic phases.

Demographic variables considered included sex, age (65–74, 75–84, and 85+ years), and insurance status. We analyzed ED characteristics and classified EDs according to hospital type and urbanity. Other relevant factors were also assessed, including ambulance use, KTAS score, arrival time, length of stay, route of arrival, and final disposition in the ED. The KTAS levels are 1–5, with 1 representing the highest and 5 indicating the lowest acuity. Route of arrival was included as it may reflect differences in care access, institutionalization, and illness severity at presentation.

### Statistical Analysis

We first conducted descriptive analyses based on patient demographics and ED visit information. We used the 2020 mid-year census population data from the Korean Statistical Information Service, provided by Statistics Korea, to calculate the annual age-standardized incidence rate of ED visits per 1,000,000 person-days. Interrupted time series analysis was applied to assess step and slope changes in monthly delirium-related ED visits from 2017–2022. The interrupted time series models incorporated the following: a dummy variable to indicate the pandemic period; an interaction term to enable step and slope changes; a covariate to capture monthly trends; and a harmonic term to adjust for seasonal effects.^24^ The total number of ED visits per month was used as the offset for the monthly estimated ED visit rate.

We stratified these analyses according to the COVID-19 pandemic period, patient age, and ED arrival route. Although COVID-19 diagnostic data were not available at the patient level, the study period was divided into three phases based on previous studies^25^,^26^: pre-pandemic (January 2017–January 2020); early pandemic (February 2020–March 2022); and late pandemic (April–December 2022). The pandemic phases (pre-, early-, and late-pandemic) were defined based on national-level changes in COVID-19 mitigation policies, particularly social distancing regulations, rather than on patient-level COVID-19 incidence or diagnoses. No direct COVID-19-confirmed case data were used in the analysis. We based temporal classification of the pandemic period on nationally recognized epidemiological turning points, beginning with the first confirmed case on January 19, 2020, and the subsequent lifting of most social isolation measures in mid-April 2022. Arrival routes were divided into two categories: direct and indirect (transferred from other hospitals). We performed all statistical analyses using SAS v9.4 (SAS Institute Inc., Cary, NC) and R statistical software v4.3.3 (The R Foundation for Statistical Computing, Vienna, Austria).

## RESULTS

**Figure 1:**
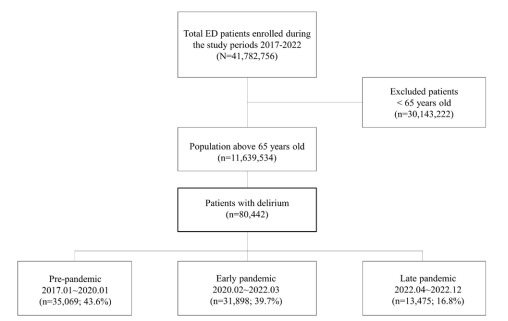
Flowchart of the study population, representing older adults with delirium. This flowchart illustrates the selection of patients ≥ 65 years of age who visited EDs in South Korea from 2017– 2022. Inclusion and exclusion criteria are detailed to define the final analytic sample used to examine trends in delirium-related ED visits during the pre-pandemic, early pandemic, and late pandemic phases. *ED*, emergency department.

The total number of older adults who visited the ED between 2017–2022 was 11,639,534. Delirium patients numbered 35,069 (43.6%) pre-pandemic, 31,898 (39.7%) during the early pandemic phase, and 13,475 (16.8%) during the late pandemic phase (Figure 1).

**Figure 2:**
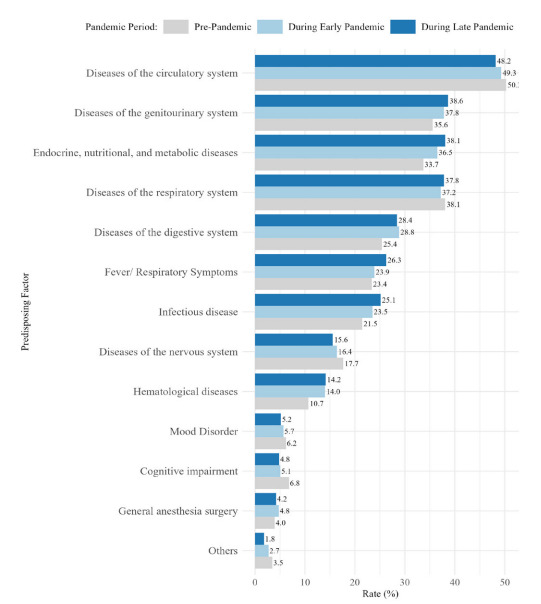
The proportion of predisposing factors in emergency department visits among older patients with delirium. This figure displays the distribution of predisposing medical conditions among older adults (≥ 65 years) who presented to EDs with delirium in South Korea. The data are stratified by pandemic phase (pre-pandemic, early pandemic, and late pandemic) to illustrate shifts in the prevalence of key contributing factors over time. *ED*, emergency department.

The number of ED patients 65–74 years of age increased steadily from 2,371 in 2017 to 3,438 in 2022. The age-standardized rate showed that there were more male than female patients with delirium (Supplementary Table 2). In general, the proportion of patients with delirium during the pandemic periods remained low compared to that during the pre-pandemic phase. The demographics showed a higher proportion of females (51.5%), 75–84 years years of age (48.3%), and National Health Insurance Service-covered patients (85.2%) who presented to the ED with delirium. Between the pre-pandemic and late pandemic phases, the proportion of KTAS level 3 patients increased (55.6% vs 57.6%), as did the length of stay (3.7 hours vs 4.9 hours) and the proportion of direct arrivals (64.5% vs 71.6%). Most patients visited the ED in the morning (31.8%). The ED disposition differed by period; the proportion of discharged patients dropped in the late pandemic phase compared to that in the pre-pandemic phase (8.4% and 5.4%, respectively). In contrast, the proportion of hospitalized patients steadily increased from 71.9% to 73.1% (Table 1). Diseases of the circulatory and respiratory systems were the main predisposing factors (Figure 2 and Supplementary Table 3) for pre-pandemic ED visits (50.3% and 38.1%, respectively).

In the early and late pandemic phases, diseases of the circulatory and genitourinary systems were the most frequent presentations (49.3% and 37.8% in the early phase, and 48.2% and 38.7% in the late phase, respectively). Overall, the incidences of infectious, hematological, metabolic, digestive system, and genitourinary system diseases, fever/respiratory symptoms, and general anesthesia surgery increased between the pre-pandemic and late-pandemic phases. Similar patterns were also observed for patients transferred from other hospitals by ambulance (Supplementary Table 4).

**Figure 3:**
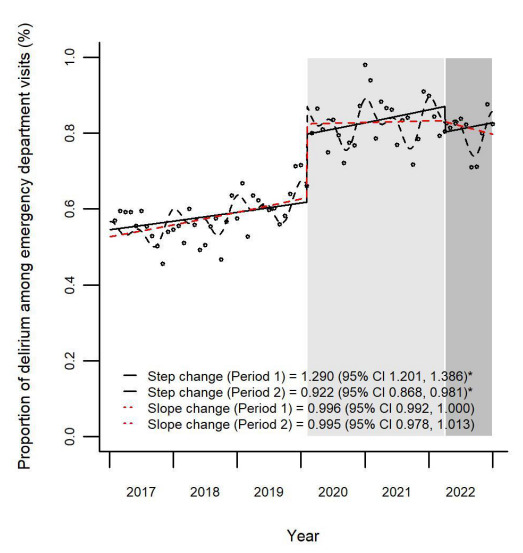
Proportion of delirium among emergency department visits before and during the COVID-19 pandemic. This figure illustrates the monthly proportion of delirium-related emergency department visits among older adults (≥65 years) in South Korea from 2017–2022. The time series is stratified into three phases: pre-pandemic (January 2017–January 2020), early pandemic (February 2020–March 2022; Period 1), and late pandemic (April 2022–December 2022; Period 2). Solid black line indicates the predicted de-seasonalized trend from a step-change regression model. Dashed black line represents the trend from a step- and slope-change model adjusted for seasonality. Dashed red line shows the de-seasonalized trend based on the full step- and slope-change regression model. Shaded bands indicate 95% CIs. *CI*, confidence intervals.

The monthly proportion of delirium among ED patients increased from 0.58% pre-pandemic to 0.83% during the early pandemic phase (reflecting a 43.1% relative increase) and then remained stable at 0.82% in the late pandemic phase. There were differences between the early pandemic (step change: relative risk (RR) 1.290, 95% CI 1.201–1.386) reflecting a 29.0% increase; slope change: RR 0.996, 95% CI 0.992–1.000); and late-pandemic phases (step change: RR 0.922, 95% CI 0.868–0.981; reflecting a 7.8% decrease; slope change: RR 0.995, 95% CI 0.978–1.013) (Figure 3).

All age groups showed a significant increase in delirium incidence during the early pandemic phase; however, only the 75+ age group showed a significant decrease during the late pandemic phase (Table 2). Both direct and indirect routes of arrival increased significantly during the early pandemic phase. In contrast, direct arrival decreased in the late pandemic phase (Tables 3).

## DISCUSSION

The number of delirium patients ≥ 65 years of age among ED visitors increased during the early phase of the COVID-19 pandemic, followed by a slight decline during the late pandemic phase. The 85+ age group showed the highest proportion of cases, whereas the 65–74 age group had the highest rate of increase. Arrivals to the ED from other facilities were significantly higher than direct arrivals, particularly of patients with surgical conditions suffering from fever and genitourinary and digestive diseases.

Prior research indicates that the overall number of ED visits with delirium has been steadily increasing for several reasons. First, the aging population is rising, leading to a higher prevalence of age-related health issues, including cognitive disorders like delirium.^27^ Second, the COVID-19 pandemic and associated isolation measures have exacerbated risk factors for delirium, especially in older adults who experience increased social isolation,^28^ reduced physical activity,^29^ and restricted access to routine mental health services, particularly those living in long-term care facilities or nursing homes.^30^ Additionally, the increased demand for medical services due to COVID-19 patient care restricted healthcare access for older adults, which may have led to an increase in patients who have not received adequate medical attention for temporary cognitive deterioration disorders, such as delirium.^31^,^32^ Lastly, heightened awareness and improved diagnostic practices may promote more frequent identification and treatment of delirium cases in emergency settings.^33^ In addition to these factors, the pandemic influenced delirium presentations through indirect mechanisms, including hospital-level constraints such as staff shortages and care delays that may have contributed to longer ED stays and more advanced disease on arrival.^34^

Several hypotheses can explain the decrease in ED visits with delirium in the late-pandemic phase. Firstly, the incidence of delirium might have decreased due to rigorous medical intervention both in community and nursing home care, although our study did not directly evaluate care quality.^35^ In South Korea, enhanced infection control protocols and increased attention to vulnerable populations during the pandemic may have contributed to more structured monitoring and prevention efforts, particularly in long-term care facilities.^36^ Secondly, families or nursing care staff living with delirium patients may have been desensitized to medical symptoms or too lethargic to let older adults visit EDs or outpatient departments; this is known as emotional exhaustion due to long-lasting COVID-19.^37^ In Korean households, where family caregivers often play a central role in elder care, prolonged caregiving burden during the pandemic may have exacerbated such emotional fatigue.^38^ This interpretation is supported by previous studies documenting increased psychological distress among caregivers during the COVID-19 pandemic. Thirdly, prolonged financial difficulties resulting from COVID-19 may have discouraged patients from seeing doctors or visiting hospitals.^39^ This trend was also observed in South Korea, where studies reported decreased healthcare use among older adults due to economic strain and fear of infection.^40^

Lastly, the incidence of social alcohol use or substance use disorder behaviors may have decreased due to the isolation/distancing strategy against COVID-19, although this hypothesis was not directly tested in our study.^41^ Post-pandemic trends need to be studied to identify the origin and context of delirium and thereby confirm these hypotheses within the Korean healthcare and social environment.^42^ While these hypotheses provide potential explanations, the current dataset did not allow for direct testing of all these mechanisms. Future studies are needed to explore these unresolved questions in greater depth.

Our finding of decreased delirium-related ED visits during the later phase of the COVID-19 pandemic in South Korea contrasts with patterns reported in other countries. For instance, a multicenter study in the United States found that 28% of older adults with COVID-19 presented to EDs with delirium, even when respiratory symptoms were absent.^43^ This discrepancy may reflect differences in healthcare access, triage protocols, hospital visitation policies, and the role of informal caregiving across systems. These international contrasts underscore the need for context-sensitive interpretation of delirium-related trends during public health emergencies.

We specifically examined the incidence of delirium, particularly among community-dwelling older adults, in this study. Delirium is characterized by a fluctuating state of confusion in which patients struggle to focus on or maintain awareness of themselves and their surroundings. Stays in the ICU and hospitalization can trigger or exacerbate delirium.^44^,^45^

In South Korea, many healthcare facilities postponed or canceled outpatient visits for patients vulnerable to the SARS-CoV-2 virus, leaving older adults defenseless against chronic illnesses. The pandemic isolation measures made it challenging to manage emergencies and maintain access to essential medical services. Routine care practices, including long-term treatment services, were interrupted by social distancing mandates.^46^ These disruptions may have contributed to the observed increase in KTAS level 1 and 2 patients and ICU admissions during the early pandemic phase, reflecting higher acuity and delayed care among older adults. While individual COVID-19 diagnosis status could not be assessed, time trends in delirium and associated variables were interpreted in the context of pandemic-related system disruptions rather than infection-related effects alone.^34^

After these restrictions were gradually lifted in the later stages of the pandemic, most delirium patients could be transferred to EDs with fewer obstacles. Interrupted time series analysis in the present study confirmed this time gap in the proportion of ED visits by patients with delirium. Behavioral restrictions, such as confinement to rooms and limited visitation, can worsen disorientation and harm cognitive function.^7^ The isolation of older adults in communities and lack of in-person care by skilled nursing facilities might have increased their risk of delirium.^11^ Government regulation or restriction regarding visits by family and acquaintances hindered the recovery of hospitalized patients.^7^ Social isolation due to distancing strategies has also been identified as a potential factor contributing to ICU delirium during the COVID-19 outbreak,^6^ with stringent isolation measures having a negative impact on the mental health of older adults, potentially exacerbating or triggering neuropsychiatric symptoms.^14^,^47^

Our results indicate that the proportion of indirect visits for delirium increased more significantly than that of direct visits during the early pandemic phase. A previous study has shown that the incidence of delirium is notably higher among long-term care residents, with these patients facing an elevated risk of functional decline, worsening dementia, and mortality. Delirium in long-term care settings also increases the likelihood of hospital admissions and contributes significantly to long-term morbidity and healthcare costs.^30^ Increased ED boarding times and constrained ICU capacity during the early pandemic may have further influenced patient disposition patterns, particularly among high-acuity cases.^48^ Our study further emphasizes that residents in long-term care facilities represent a group particularly vulnerable to delirium.

Given these challenges, establishing proper referral systems during a pandemic is required, especially to manage an increase in ED visits by older delirium patients. Social isolation and aging can exacerbate mental symptoms; therefore, proactive community-based strategies are essential. Firstly, enhancing accessible mental health support and regular check-ins for older adults during a pandemic can mitigate the negative impacts of isolation and reduce the risk of delirium.^49^ Community health programs as preventive measures should be implemented to promote physical activity, social engagement, and cognitive stimulation, all of which have been shown to lower delirium risk.^50^ Additionally, establishing telehealth services and expanding home healthcare resources for older adults, including those in long-term care facilities, would allow timely intervention and reduce unnecessary ED visits.^51^ Adequate training of healthcare workers, family members, and caregivers to recognize early delirium symptoms is also essential. Early detection and proper management can significantly alleviate the symptoms of delirium and reduce ED reliance.^52^ A well-coordinated response within communities will be critical for proactively addressing delirium in older adults and building resilience for future pandemics.

The proportion of delirium identified in this study was lower than prevalence estimates reported in prior studies, which have ranged from 7–30% among older ED patients based on structured screening tools.^2^,^3^ This discrepancy is likely attributable to the use of diagnostic codes from administrative data rather than prospective clinical assessments. Delirium is frequently under-recognized in emergency care, particularly in patients with hypoactive symptoms, and is often undercoded in discharge summaries and hospital records. Nonetheless, the observed demographic and clinical characteristics of this study, such as predominance among individuals aged 75–84 years, females, and patients with circulatory or respiratory conditions, are consistent with previous papers.^53^,^54^

Internationally, the prevalence and documentation of delirium vary widely depending on healthcare infrastructure, assessment practices, and clinical priorities. A recent meta-analysis reported that delirium prevalence in COVID-19 patients ranged from 11–80% across countries,^55^ suggesting substantial variability in recognition and reporting. In addition, a global survey across 44 countries revealed that only two-thirds of hospital units used validated delirium assessment tools, and the presence of formal delirium protocols varied considerably by region.^56^ These findings underscore that the patterns observed in South Korea, such as a decrease in delirium-related ED visits during the pandemic may be shaped by country-specific factors such as triage protocols, long-term care capacity, family caregiving roles, and cultural attitudes toward emergency care. Thus, international comparisons must be interpreted in the context of these systemic and cultural differences.

## LIMITATIONS

Our study had several limitations. Firstly, delirium was defined solely based on diagnostic codes, which may have decreased the precision in accurately identifying delirium cases. Additionally, although COVID-19 is associated with an increased incidence of delirium symptoms, this trend was not specifically analyzed among COVID-19-positive patients. Although the study period was categorized into pandemic phases, individual-level COVID-19 diagnosis data or incidence rates were not incorporated into the analysis. This may have limited the precision in attributing observed trends directly to the pandemic’s clinical impact. Our phase classification was instead based on broad policy timelines (eg, implementation of social distancing), which may not fully reflect the epidemiological burden of COVID-19 during each period. Our use of visit-level data also introduced the potential for duplicate visits, which may not accurately reflect the actual incidence of delirium.

Furthermore, social distancing measures varied slightly by region, but a detailed regional analysis was not conducted. Lastly, although the group of patients from nursing homes was not directly investigated, an indirect assessment of nursing facility transfers was conducted, defining indirect arrivals as those involving private ambulances. Another key limitation of this study is the absence of patient-level, COVID-19 diagnostic information in the NEDIS dataset. As a result, we were unable to directly assess delirium cases attributable to confirmed infection. Instead, we analyzed temporal trends in the context of broader system-level disruptions, such as social distancing policies and ED utilization patterns.

In addition, several contextual factors related to the pandemic itself could not be fully captured in our data. Operational changes in hospitals such as staffing shortages, resource reallocation, and increased length of ED stay may have influenced the detection and documentation of delirium, as well as patterns of ED use. Furthermore, due to the limitations of administrative data, our variable selection did not include important psychosocial or clinical factors such as baseline cognitive status, caregiver stress, or social isolation. These unmeasured variables may have affected not only the incidence of delirium, but also patient or caregiver decision-making regarding ED visits. As a result, while our findings offer meaningful insights into national-level trends, their utility and generalizability may be limited by these data constraints.

Despite these limitations, this study is the first in South Korea to investigate the trend in ED visits for delirium among community-dwelling older adults, and provides critical insights into delirium occurrence during the pandemic at a national level. A comprehensive picture of delirium trends across diverse populations during the pandemic was obtained using nationwide data. Additionally, our study examined the importance of a time-framed strategy for medical intervention. More precise and categorical interventions are required to deal with psychiatric emergencies, such as delirium and other mental and physical conditions, especially during pandemics.

## CONCLUSION

We analyzed trends in delirium-related ED visits among older adults in South Korea before and during the COVID-19 pandemic, with a focus on the phases of social distancing. A notable 7.8% decrease in visits was identified during the late pandemic period, and key risk factors were highlighted, particularly among adults aged 65–74 (40.6% increase during the early pandemic) and those referred from institutions (27.5% increase during the early pandemic). These findings help elucidate the pandemic’s impact on mental health and may inform targeted interventions and public health responses in similar healthcare systems or settings. While this study was based on national data from South Korea, the observed trends may offer preliminary insights for emergency care systems in other settings, particularly those with aging populations or facing similar healthcare disruptions during public health emergencies. Further research is needed to evaluate whether these patterns are consistent across different healthcare environments and to better understand their implications for emergency medicine practice globally.

## Supplementary Information



## Figures and Tables

**Table 1:** Demographics of visits to emergency departments by older adults with delirium before and during the COVID-19 pandemic.

Variable	Category	Total N (%)	Pre-pandemic n (%)	Early pandemic n (%)	Late Pandemic n (%)
Total		80,442 (100.0)	35,069 (43.6)	31,898 (39.7)	13,475 (16.8)
Sex	Male	39,011 (48.5)	16,974 (48.4)	15,502 (48.6)	6,535 (48.0)
	Female	41,431 (51.5)	18,095 (51.6)	16,396 (51.4)	6,940 (51.5)
Age group	65–74	16,791 (20.87)	7,457 (21.26)	6,623 (20.76)	2,711 (20.1)
	75–84	38,842 (48.3)	17,569 (50.1)	15,120 (47.4)	6,153 (45.66)
	85+	24,809 (30.8)	10,043 (28.6)	10,155 (31.8)	4,611 (34.2)
Insurance	NHI	68,522 (85.2)	29,588 (84.4)	27,309 (85.6)	11,625 (86.3)
	Medical aid	9,679 (12.0)	4,344 (12.4)	3,766 (11.8)	1,569 (11.6)
	No insurance	412 (0.5)	207 (0.6)	153 (0.5)	52 (0.4)
	Other or UK	1,829 (2.3)	930 (2.7)	670 (2.1)	229 (1.7)
Ambulance use	Yes	54,467 (67.7)	23,323 (66.5)	22,001 (69.0)	9,143 (67.9)
Length of Stay	Median (IQR)	4.28 (5.4)	3.73 (4.6)	4.75 (6.2)	4.85 (5.3)
	< 6 hours	52,206 (64.9)	24,839 (70.8)	19,161 (60.1)	8,206 (60.9)
	> 6 hours	28,236 (35.1)	10,230 (29.2)	12,737 (39.9)	5,269 (39.1)
Hospital	Regional emergency medical center	19,519 (24.3)	8,129 (23.2)	7,880 (24.7)	3,510 (26.1)
	Local emergency medical center	43,850 (54.5)	18,069 (51.5)	18,427 (57.8)	7,354 (54.6)
	Local emergency medical institution	17,073 (21.2)	8,871 (25.3)	5,591 (17.5)	2,611 (19.4)
KTAS^a^	1 (resuscitation)	1,964 (3.1)	788 (3.0)	842 (3.2)	334 (3.1)
	2 (emergent)	10,784 (17.0)	4,487 (17.1)	4,391 (16.7)	1,906 (17.5)
	3 (urgent)	35,576 (56.2)	14,563 (55.6)	14,756 (56.1)	6,257 (57.6)
	4 (less urgent)	13,354 (21.1)	5,615 (21.5)	5,619 (21.4)	2,120 (19.5)
	5 (non-urgent)	1,661 (2.6)	715 (2.7)	699 (2.7)	247 (2.3)
	UK	5 (0.0)	5 (0.0)	0 (0.0)	0 (0.0)
Urbanity	Urban	78,183 (97.2)	34,036 (97.1)	31,020 (97.3)	13,127 (97.4)
	Rural	2,259 (2.8)	1,033 (3.0)	878 (2.8)	348 (2.6)
Time of Presentation	Morning (6am–12pm)	25,587 (31.8)	10,857 (31.0)	10,382 (32.6)	4,348 (32.3)
	Afternoon(12pm–6 pm)	32,397 (40.3)	14,315 (40.8)	12,714 (39.9)	5,368 (39.8)
	Nighttime (6pm–1am)	17,965 (22.33)	7,909 (22.6)	7,045 (22.1)	3,011 (22.4)
	Dawn (1am–6am)	4,493 (5.6)	1,988 (5.7)	1,757 (5.5)	748 (5.6)
Route of arrival	Direct	56,696 (70.5)	23,958 (68.32)	22,837 (71.59)	9,901 (73.48)
	Indirect^b^	21,269 (26.4)	10,216 (29.1)	7,942 (24.9)	3,111 (23.1)
	Other^c^ or UK	2,477 (3.1)	895 (2.6)	1,119 (3.51	463 (3.4)
ED disposition	Discharged	5,371 (6.7)	2,949 (8.4)	1,696 (5.32)	726 (5.39)
	Transferred	776 (0.96)	469 (1.34)	224 (0.7)	83 (0.6)
	Hospitalization	58,213 (72.4)	25,204 (71.9)	23,160 (72.6)	9,849 (73.1)
	ICU admission	16,028 (20.0)	6,422 (18.3)	6,798 (21.3)	2,808 (20.8)
	Died	27 (0.0)	14 (0.0)	7 (0.0)	6 (0.0)
	Other or UK	27 (0.0)	11 (0.0)	13 (0.0)	3 (0.0)

a Only Level 1 (regional emergency medical center) and Level 2 (local emergency medical center) EDs were included due to their low rate of missing data.

b Indirect includes transfer from other hospital.

c Other includes refer from outpatient clinics.

This table presents the demographic and clinical characteristics of older adults with delirium-related emergency department visits in South Korea, stratified by pandemic phase to assess trends before and during the COVID-19 pandemic.

*UK*, unknown; *ICU*, intensive care unit; *ED*, emergency department; *IQR*, interquartile range; *NHI*, National Health Insurance.

**Table 2:** Association between the COVID-19 pandemic and changes in emergency department visits for delirium by age group: a comparison of pre-pandemic, early, and late pandemic periods.

Age group	Period (Ref. Pre-pandemic)	Mean Monthly proportion of Delirium to the total ED visit		Relative risk (95% CI)	
		Before	During	Step change	Slope change
65–74	Period 1	0.28%	0.38%	1.406 (1.264, 1.564)^*^	1.001 (0.995, 1.007)
	Period 2		0.37%	0.957 (0.873, 1.049)	1.000 (0.973, 1.028)
75–84	Period 1	0.71%	0.99%	1.289 (1.196, 1.389)^*^	0.996 (0.992, 1.000)
	Period 2		0.96%	0.912 (0.855, 0.972)^*^	0.987 (0.969, 1.006)
85+	Period 1	1.13%	1.57%	1.232 (1.132, 1.342)^*^	0.995 (0.990, 0.999)^*^
	Period 2		1.54%	0.900 (0.837, 0.968)^*^	1.001 (0.981, 1.023)

This table presents the mean monthly proportion of delirium-related emergency department visits and corresponding relative risks (RR) for each age group (65–74, 75–84, and ≥ 85 years), comparing the pre-pandemic, early pandemic (Period 1: February 2020–March 2022), and late pandemic (Period 2: April 2022–December 2022) periods in South Korea. Step and slope changes were estimated using interrupted time series analysis with the pre-pandemic period as the reference. An asterisk (*) indicates stasignificance at P < .05.

*Ref*, reference category; *ED*, emergency department; *RR*, relative risk.

**Table 3:** Association between the COVID-19 pandemic and changes in emergency department visits for delirium by route of arrival: a comparison of pre-pandemic, early-, and late-pandemic periods.

Route of arrival	Period (Ref. Pre-pandemic)	Mean monthly proportion of delirium to the total ED visit (%)		Relative risk (95% CI)	
		Before	During	Step change	Slope change
Direct	Period 1	0.47%	0.70%	1.275 (1.184, 1.373)^*^	0.996 (0.992, 1.001)
	Period 2		0.69%	0.910 (0.855, 0.969)^*^	0.994 (0.976, 1.012)
Indirect	Period 1	1.18%	1.61%	1.334 (1.238, 1.438)^*^	0.996 (0.992, 1.000)
	Period 2		1.62%	0.995 (0.933, 1.060)	0.989 (0.971, 1.008)

This table shows the mean monthly proportion of delirium-related emergency department visits and corresponding relative risks for patients categorized by route of arrival: direct (eg, self-transport, public ambulance) and indirect (eg, transfer from another hospital via private ambulance). The data compare the pre-pandemic, early pandemic (Period 1: February 2020–March 2022), and late pandemic (Period 2: April 2022–December 2022) phases. Step and slope changes were estimated using interrupted time series analysis, with the pre-pandemic period as the reference. An asterisk (*) indicates statistical significance at *P* < .05.

*Ref*, reference category; *ED*, emergency department; *RR*, relative risk.
