# Supplementary material for: Trends in Proportion of Delirium Among Older Emergency Department Patients in South Korea, 2017–2022
Source: West J Emerg Med. 2025 Nov 26;26(6):1744–54. doi: 10.5811/westjem.41507 (PMC12698174; doi:10.5811/westjem.41507)
Supplement: Supplementary file 1 [file wjem-26-1744-s001.docx]

**Trends in proportion of delirium among older emergency department patients in South Korea, 2017-2022.**

**Supplementary Table 1.** International classification of diseases diagnosis code and UMLS codes for predisposing factors.

**Supplementary Table 2.** Annual incidence for ED visit counts and age-standardized rate with delirium in older population.

**Supplementary Table 3.** Predisposing factors for ED visits with delirium among older population.

**Supplementary Table 1.** International classification of diseases diagnosis code and UMLS codes for predisposing factors.

| **Predisposing factors** | **ICD-10 codes or UMLS codes** |
| --- | --- |
| Infectious disease | A00-B99 |
| Hematological diseases | D50-D89 |
| Endocrine, nutritional, and metabolic diseases | E00-E90 |
| Diseases of the nervous system | G00-G99 |
| Diseases of the circulatory system | I00-I99 |
| Diseases of the respiratory system | J00-J99 |
| Diseases of the digestive system | K00-K93 |
| Diseases of the genitourinary system | N00-N99 |
| Cognitive impairment | F02-F03 |
| Mood Disorder | F30-F39 |
| Psychotic Disorder | F20-F29 |
| Vision impairment | H25-H26, H54 |
| Hearing impairment | H90-H91 |
| Fever/ Respiratory Symptoms | C0015967, C0015970, C0085594, C0239575, C0277799, C1277295, C0010200, C0010201, C0234866, C0239134, C0425508, C0850149, C1277295, C0239134, C0241235, C0241235, C0476275, C0476276, C0476277, C0555056, C0577978, C0577979, C0577982, C0242429, C0013404, C0231803, C0231804, C0231805, C0231806, C0231807, C0743330 |
| Drug addiction | T36-T50 |

**Supplementary Table 2.** Annual incidence for ED visit counts and age-standardized rate with delirium in older population.

| ED visits | Total | Overall | Year | | | | | |
| --- | --- | --- | --- | --- | --- | --- | --- | --- |
|  |  |  | 2017 | 2018 | 2019 | 2020 | 2021 | 2022 |
|  |  | N | N | N | N | N | N | N |
| Age group | 65-74 | 16,791 | 2,371 | 2,305 | 2,511 | 2,853 | 3,313 | 3,438 |
|  | 75-84 | 38,842 | 5,028 | 5,581 | 6,344 | 6,758 | 7,331 | 7,800 |
|  | 85+ | 24,809 | 2,681 | 3,085 | 3,862 | 4,318 | 5,060 | 5,803 |
| Sex | Male | 39,011 | 4,838 | 5,330 | 6,150 | 6,758 | 7,680 | 8,255 |
|  | age-standardized rate | 10.2 | 13.7 | 13.4 | 15.0 | 18.8 | 20.5 | 19.2 |
|  | Female | 41,431 | 5,242 | 5,641 | 6,567 | 7,171 | 8,024 | 8,786 |
|  | age-standardized rate | 7.8 | 11.5 | 11.21 | 12.3 | 16.2 | 16.6 | 15.8 |

Note: Age-standardized rate is the annual incidence rate of ED visits per 1,000,000 persons.

**Supplementary Table 3.** Predisposing factors for ED visits with delirium among older population.

|  | Total | Pre-pandemic | Early pandemic | Late pandemic |  |
| --- | --- | --- | --- | --- | --- |
| Predisposing factors | N (%) | N (%) | N (%) | N (%) | P-value |
| Infectious disease | 18,415 (22.9) | 7,527 (21.5) | 7,500 (23.5) | 3,388 (25.1) | <0.001 |
| Hematological diseases | 10,118 (12.6) | 3,748 (10.7) | 4,462 (14.0) | 1,908 (14.2) | <0.001 |
| Endocrine, nutritional, and metabolic diseases | 28,585 (35.5) | 11,814 (33.7) | 11,638 (36.5) | 5,133 (38.1) | <0.001 |
| Diseases of the nervous system | 13,516 (16.8) | 6,198 (17.7) | 5,223 (16.4) | 2,095 (15.6) | <0.001 |
| Diseases of the circulatory system | 39,838 (49.5) | 17,627 (50.3) | 15,718 (49.3) | 6,493 (48.2) | <0.001 |
| Diseases of the respiratory system | 30,298 (37.7) | 13,348 (38.1) | 11,855 (37.2) | 5,095 (37.8) | 0.053 |
| Diseases of the digestive | 21,926 (27.3) | 8,899 (25.4) | 9,196 (28.8) | 3,831 (28.4) | <0.001 |
| Diseases of the genitourinary system | 29,741 (37.0) | 12,475 (35.6) | 12,058 (37.8) | 5,208 (38.7) | <0.001 |
| Cognitive impairment | 4,650 (5.8) | 2,385 (6.8) | 1,616 (5.1) | 649 (4.8) | <0.001 |
| Mood Disorder | 4,687 (5.8) | 2,170 (6.2) | 1,819 (5.7) | 698 (5.2) | <0.001 |
| Psychotic Disorder | 1,388 (1.7) | 754 (2.2) | 506 (1.6) | 128 (1.0) | <0.001 |
| Vision impairment | 233 (0.3) | 137 (0.4) | 75 (0.3) | 21 (0.2) | <0.001 |
| Hearing impairment | 159 (0.2) | 80 (0.2) | 60 (0.2) | 19 (0.1) | 0.136 |
| Fever/ Respiratory Symptoms^a^ | 15,260 (24.1) | 6,123 (23.4) | 6,284 (23.9) | 2,853 (26.3) | <0.001 |
| General anesthesia surgery | 3,471 (4.3) | 1,384 (4.0) | 1,519 (4.8) | 568 (4.2) | <0.001 |
| Drug addiction | 543 (0.7) | 246 (0.7) | 222 (0.7) | 75 (0.6) | 0.183 |

^a^Only Level 1 (Regional emergency medical center) and Level 2 (Local emergency medical center) EDs were included due to their low rate of missing data.

**Supplementary Table 4.** Predisposing factors for delirium-related ED visits among older population by arrival route.

|  | Pre-pandemic | | | Early pandemic | | | Late pandemic | | | |
| --- | --- | --- | --- | --- | --- | --- | --- | --- | --- | --- |
|  | Indirect^a^ | Direct |  | Indirect^a^ | Direct |  | Indirect^a^ | Direct |  |  |
| Predisposing factors | N (%) | N (%) | p value | N (%) | N (%) | p value | N (%) | N (%) | p value |  |
| Older age (Age group 85y+) | 2,643 (25.9) | 7,222 (30.1) | <0.001 | 2,284 (28.8) | 7,613 (33.3) | <0.001 | 979 (31.5) | 3,508 (35.4) | <0.001 |  |
| Male group | 5,133 (50.2) | 11,361 (47.4) | <0.001 | 4,021 (50.6) | 10,885 (47.7) | <0.001 | 1,596 (51.3) | 4,690 (47.4) | <0.001 |  |
| Infectious disease | 2,553 (25.0) | 4,770 (19.9) | <0.001 | 2,015 (25.4) | 5,183 (22.7) | <0.001 | 902 (29.0) | 2,370 (23.9) | <0.001 |  |
| Hematological diseases | 1,201 (11.8) | 2,434 (10.2) | <0.001 | 1,288 (16.2) | 3,000 (13.1) | <0.001 | 523 (16.8) | 1,303 (13.2) | <0.001 |  |
| Endocrine, nutritional, and metabolic diseases | 3,568 (34.93) | 7,910 (33.02) | <0.001 | 2,897 (36.48) | 8,320 (36.43) | 0.954 | 1,239 (39.83) | 3,718 (37.55) | 0.024 |  |
| Diseases of the nervous system | 1,857 (18.2) | 4,192 (17.5) | 0.1356 | 1,239 (15.6) | 3,787 (16.6) | 0.043 | 484 (15.6) | 1,539 (15.5) | 1.0000 |  |
| Diseases of the circulatory system | 5,685 (55.7) | 11,456 (47.8) | <0.001 | 4,145 (52.2) | 10,975 (48.1) | <0.001 | 1,618 (52.0) | 4,644 (46.9) | <0.001 |  |
| Diseases of the respiratory system | 4,423 (43.3) | 8,605 (35.9) | <0.001 | 3,300 (41.6) | 8,137 (35.6) | <0.001 | 1,276 (41.0) | 3,637 (36.7) | <0.001 |  |
| Diseases of the digestive | 2,797 (27.4) | 5,890 (24.6) | <0.001 | 2,551 (32.1) | 6,321 (27.7) | <0.001 | 993 (31.9) | 2,734 (27.6) | <0.001 |  |
| Diseases of the genitourinary system | 3,837 (37.6) | 8,323 (34.7) | <0.001 | 3,083 (38.8) | 8,545 (37.4) | 0.027 | 1,269 (40.8) | 3,755 (37.9) | 0.005 |  |
| Cognitive impairment | 728 (7.1) | 1,609 (6.7) | 0.176 | 452 (5.7) | 1,085 (4.8) | 0.001 | 154 (5.0) | 471 (4.8) | 0.696 |  |
| Mood Disorder | 564 (5.5) | 1,553 (6.5) | <0.001 | 434 (5.5) | 1,314 (5.8) | 0.352 | 140 (4.5) | 532 (5.4) | 0.061 |  |
| Psychotic Disorder | 218 (2.1) | 521 (2.2) | 0.844 | 133 (1.7) | 363 (1.6) | 0.640 | 21 (0.7) | 102 (1.0) | 0.093 |  |
| Vision impairment | 42 (0.4) | 89 (0.4) | 0.568 | 14 (0.2) | 58 (0.3) | 0.280 | 5 (0.2) | 15 (0.2) | 1.000 |  |
| Hearing impairment | 23 (0.2) | 56 (0.2) | 1.000 | 14 (0.2) | 44 (0.2) | 0.881 | 1 (0.0) | 16 (0.2) | 0.092 |  |
| Fever/ Respiratory Symptoms^b^ | 2,442 (28.7) | 3,491 (20.7) | <0.001 | 1,795 (26.0) | 4,227 (23.0) | <0.001 | 741 (27.7) | 1,996 (25.7) | 0.040 |  |
| General anesthesia surgery | 520 (5.1) | 828 (3.5) | <0.001 | 527 (6.6) | 941 (4.1) | <0.001 | 183 (5.9) | 369 (3.7) | <0.001 |  |
| Drug addiction | 48 (0.5) | 198 (0.8) | <0.001 | 37 (0.5) | 182 (0.8) | 0.003 | 6 (0.2) | 69 (0.7) | 0.002 |  |

^a^Indirect indicates transfer from other hospital.

^b^Only Level 1 (Regional emergency medical center) and Level 2 (Local emergency medical center) EDs were included due to their low rate of missing data
